# Tonsils at Telethon: developing a standardised collection of tonsil photographs for group A streptococcal (GAS) research

**DOI:** 10.3389/fped.2024.1367060

**Published:** 2024-04-25

**Authors:** Marianne J. Mullane, Hannah M. Thomas, Jonathan R. Carapetis, Catalina Lizama, Wesley Billingham, Matthew N. Cooper, Christine Everest, Claudia R. Sampson, Nelly Newall, Sarah Pearce, Francis Lannigan, Eamonn McNulty, Rebecca Cresp, Ariel O. Mace, Tina Barrow, Asha C. Bowen

**Affiliations:** ^1^ Wesfarmers Centre of Vaccines and Infectious Diseases, Telethon Kids Institute, University of Western Australia, Perth, WA, Australia; ^2^ Department of Infectious Diseases, Perth Children’s Hospital, Perth, WA, Australia; ^3^ School of Medicine, The University of Notre Dame Australia, Fremantle, WA, Australia

**Keywords:** group A streptococcus (GAS), *Streptococcus pyogenes*, pharyngitis, acute rheumatic fever, photographs, tonsils

## Abstract

**Introduction:**

Group A streptococcus (GAS) infections, such as pharyngitis and impetigo, can lead to rheumatic fever and rheumatic heart disease (RHD). Australian Aboriginal and Torres Strait Islander populations experience high rates of RHD and GAS skin infection, yet rates of GAS pharyngitis are unclear. Anecdotally, clinical presentations of pharyngitis, including tonsillar hypertrophy and sore throat, are uncommon. This study aimed to develop a standardised set of tonsil photographs and determine tonsil size distribution from an urban paediatric population.

**Methods:**

A prospective cohort of children aged 3–15 years were recruited at the public events “Discover Day” and “Telethon Weekend” (October 2017) in Perth, Western Australia, Australia. Tonsil photographs, symptomatology, and GAS rapid antigen detection tests (RADT) were collected. Tonsil size was graded from the photographs using the Brodsky Grading Scale of tonsillar hypertrophy (Brodsky) by two independent clinicians, and inter-rater reliability calculated. Pharyngitis symptoms and GAS RADT were correlated, and immediate results provided.

**Results:**

Four hundred and twenty-six healthy children participated in the study over three days. The median age was seven years [interquartile range (IQR) 5.9–9.7 years]. Tonsil photographs were collected for 92% of participants, of which 62% were rated as good-quality photographs and 79% were deemed of adequate quality for assessment by both clinicians. When scored by two independent clinicians, 57% received the same grade. Average Brodsky grades (between clinicians) were 11%, 35%, 28%, 22% and 5% of grades 0,1,2,3 and 4, respectively. There was moderate agreement in grading using photographs, and minimal to weak agreement for signs of infection. Of 394 participants, 8% reported a sore throat. Of 334 GAS RADT performed, <1% were positive.

**Discussion:**

We report the first standardised use of paediatric tonsil photographs to assess tonsil size in urban-living Australian children. This provides a proof of concept from an urban-living cohort that could be compared with children in other settings with high risk of GAS pharyngitis or rheumatic fever such as remote-living Australian Indigenous populations.

## 1 Introduction

Acute rheumatic fever (ARF) is the most common cause of acquired heart disease in children worldwide in low resource settings (1, 2). Rates of ARF in Australian Aboriginal and Torres Strait Islander peoples (hereafter respectfully referred to as Indigenous) are amongst the highest reported in the world, at 374–508 per 100,000 aged 5–15 years (3–5). The most serious sequelae of ARF, rheumatic heart disease (RHD), occurs in 42%–60% of people with a prior history of ARF (3–6). Indigenous populations in Northern Australia have the highest reported rates of mortality from ARF or RHD worldwide (23.8 per 100,000) (3).

ARF is an autoimmune-mediated response that typically occurs 2–3 weeks after a group A streptococcal (GAS) throat infection (pharyngitis) or skin infection (impetigo) (7). In remote-living Australian Indigenous children with high RHD rates, GAS skin infections are endemic (8) whilst pharyngitis burden is uncommon (9). The reported incidence of sore throat is low at approximately 8 episodes/100 children/year [95% confidence interval (CI), 4–15 episodes per 100 person-years], and for symptomatic GAS pharyngitis, the reported incidence density is 0 (10). In contrast, a small study of 151 children with ARF found that 25% of remote-living Indigenous children had a recent sore throat preceding ARF diagnosis (11). Anecdotally, clinicians have reported that tonsillar hypertrophy (large/swollen tonsils) and sore throat presentations appear to be uncommon in Australian Indigenous children, compared to non-Indigenous Australian children.

Other research studies conducted by this team document the concurrent burden of GAS throat and skin infections in Australian Indigenous populations and assess the hypothesis that Australian Indigenous children do not commonly report sore throat or present with large tonsils (12). Most of the literature reporting on tonsil size in children is in the context of anthropometrics and sleep-disordered breathing, and preoperative clinical decision-making for tonsillectomies in clinical/hospital settings (13). Akcay and colleagues (14) developed a tonsil size distribution curve (via physical examination and grading) from 1,784 school children living in the city of Denizli, Turkey (14). Tonsil size distribution in Australian urban or remote-living children has not been documented previously. Further, using a photograph to illustrate tonsil size and hypertrophy has not been previously trialed in children, although some research has been conducted with adults (15). Medical photography can be a useful clinical tool that increases documentation, and improves both doctor-patient and inter-provider communication in remote Telehealth consultation (16). We developed a protocol for photography of the tonsils that could be used in research studies in remote settings as part of GAS pharyngitis assessments. Using this protocol, the project aimed to develop a standardised set of tonsil photographs to determine tonsil size distribution from an urban population of healthy children, to later compare with those collected from remote Indigenous children. The secondary aims were to document the presence of sore throat symptoms in participants, and where feasible, assess for the presence of GAS in the throat using a Rapid Antigen Detection Test (RADT). The secondary aims were not essential to the project but were included to enhance the participants' interaction with public science activities (described further in the methods).

## 2 Materials & methods

### 2.1 Study setting and design

This was a prospective, cross-sectional study that was embedded into three days of public science events: Telethon Kids Institute “Discover Day” and “Channel 7 Perth's Telethon Weekend”, located in Perth, Western Australia, Australia in October 2017.

### 2.2 Recruitment and selection criteria

Recruitment was completed using convenience sampling. Children aged 3–15 years attending the public science events and who were accompanied by a parent or legal guardian were eligible to participate. Children were excluded if they had undergone a tonsillectomy in the preceding month (due to the potential bleeding risk from throat swab collection).

### 2.3 Online informed consent

Due to the nature of the study being incorporated into public science events attracting the attendance of hundreds of families, online informed consent (using a REDCap database) (17) was used to reduce the paperwork burden on families. Copies of the online consent forms were emailed to participants upon request.

### 2.4 Training of research staff

Forty staff and students from Telethon Kids Institute and Perth Children's Hospital attended a one-hour training session on study consent, photography, and data collection procedures according to standard operating procedures. These staff and students carried out data collection during the public science events.

### 2.5 Data collection

As this study was incorporated into public science events that were attended by hundreds of families, data collection methods needed to be fast-paced and engaging. Children were invited to become “Tonsil Detectives”, and data collection involved three main activities:

(a) “Take a tonsil selfie” (the primary objective of the study: collection of tonsil photographs). Photographs were taken by trained research staff using the iPhone 7 Plus and standard operating procedures (see Supplementary Figure S1). Multiple tonsil photographs were taken for each individual, with the highest quality photograph selected by the study chief investigator for clinical assessment. Two independent clinicians then assessed the chosen photographs and rated quality of the photograph (good or poor), assessed signs of infection (presence or absence of swelling, pus, and/or redness) and graded tonsil size using the Brodsky grading scale (Supplementary Figure S2), a standardised and reproducible measurement often used in paediatrics (18–20). Due to the potential subjectivity of this assessment, this data was analysed for inter-observer reliability, and a mean was calculated to report the tonsil size results.
(b) “Tell us about your tonsils”: A brief checklist completed with the child and/or parent to assess for self-reported symptoms of pharyngitis. This checklist was created with input from paediatric infectious disease specialists and based on their typical symptomology questions for assessing GAS pharyngitis in clinical care. Basic demographics were also recorded.
(c) “Test your tonsils”: RADT for GAS. This was an additional optional data collection activity for those interested and consented. Tonsillar swabs were collected by passing a swab stick over the tonsils, as per standard operating procedures. For detection of GAS, the BIOMÉRIEUX bioNexia® Strep A Plus RADT kits (21) were used, which engaged the participants with hands on use of diagnostic tests whilst also providing a result within five minutes.

Participants with sore throat symptoms and a positive RADT result (if collected), were provided with a letter to take to their doctor to be further assessed for treatment. Participants with a positive RADT in the absence of sore throat symptoms, were recommended to visit their doctor only if they developed other signs of sickness or infection (e.g., fever).

### 2.6 Definitions

For the independent clinicians who assessed the tonsil photographs, a good quality photograph was defined as one where tonsils could be clearly seen and were easy to assess. Poor quality photographs were defined as those where image quality was suboptimal (out of focus, unclear, too dark/bright), therefore making assessment of tonsils difficult, but enough information was present to make a valid attempt. Clinicians did not grade or assess photographs where suboptimal quality made a valid attempt not possible.

### 2.7 Data entry and analysis

All data were entered in real-time into online case report forms (CRFs) developed in a REDCap database (17). Inter-rater reliability metrics were calculated, the primary reported statistic being the squared-weighted κ; equal-weighted κ is also reported for comparison. κ statistics were interpreted in line with definitions provided by McHugh (22). Linear regression was used (following graphical review of residuals to assess modelling assumptions) to quantify the relationship between age and mean (of the two assessors) assigned grade; the beta coefficient (and 95% confidence interval) for age is also reported. All analysis was carried out in R (23, 24).

### 2.8 Ethical considerations

Informed consent was documented electronically from all participant's parent and/or legal guardian, who were required to be present at the public science activities for their child to participate. Ethics approval was obtained from the University of Western Australia Human Research Ethics Office (RA/4/1/9277).

## 3 Results

### 3.1 Demographics

Four hundred and twenty-six children participated in the study over three days. Demographic information was collected for 393 (92.3%) participants. The median age was 7.4 years [interquartile range (IQR) 5.9–9.7]. Two hundred and thirty-nine (56.1%) of the participants were female, 261 (61.3%) identified as Caucasian and eight (1.9%) identified as Indigenous (see Supplementary Table S1).

### 3.2 Quality of photographs

Tonsil photographs were collected for 393/426 (92.3%) participants of which 391 (99.5%) were reviewed by the two independent clinicians for quality. There was weak agreement (*k* = 0.42, *p *< .001) between clinicians on the quality of photographs. The two clinicians assessed 242 (61.6%) of the tonsil photographs as good-quality and 150 (38.2%) as poor-quality photographs. The clinicians disagreed on 107 (27.2%).

### 3.3 Tonsil photographs (“take a tonsil selfie”)

#### 3.3.1 Agreement between reviewers of photographs assessed

There was moderate agreement (*k* = 0.73, *p *< .001) between clinicians on whether photographs could be assessed for Brodsky grading. Fifty-two photographs (13.2%) were deemed un-assessable by both clinicians. Clinician one assessed 13 photographs that clinician two deemed un-assessable, and clinician two assessed 17 that clinician one deemed un-assessable, for a total of 311/393 (79.1%) which were assessed by both clinicians.

#### 3.3.2 Tonsil size (Brodsky grade)

**Figure 1:**
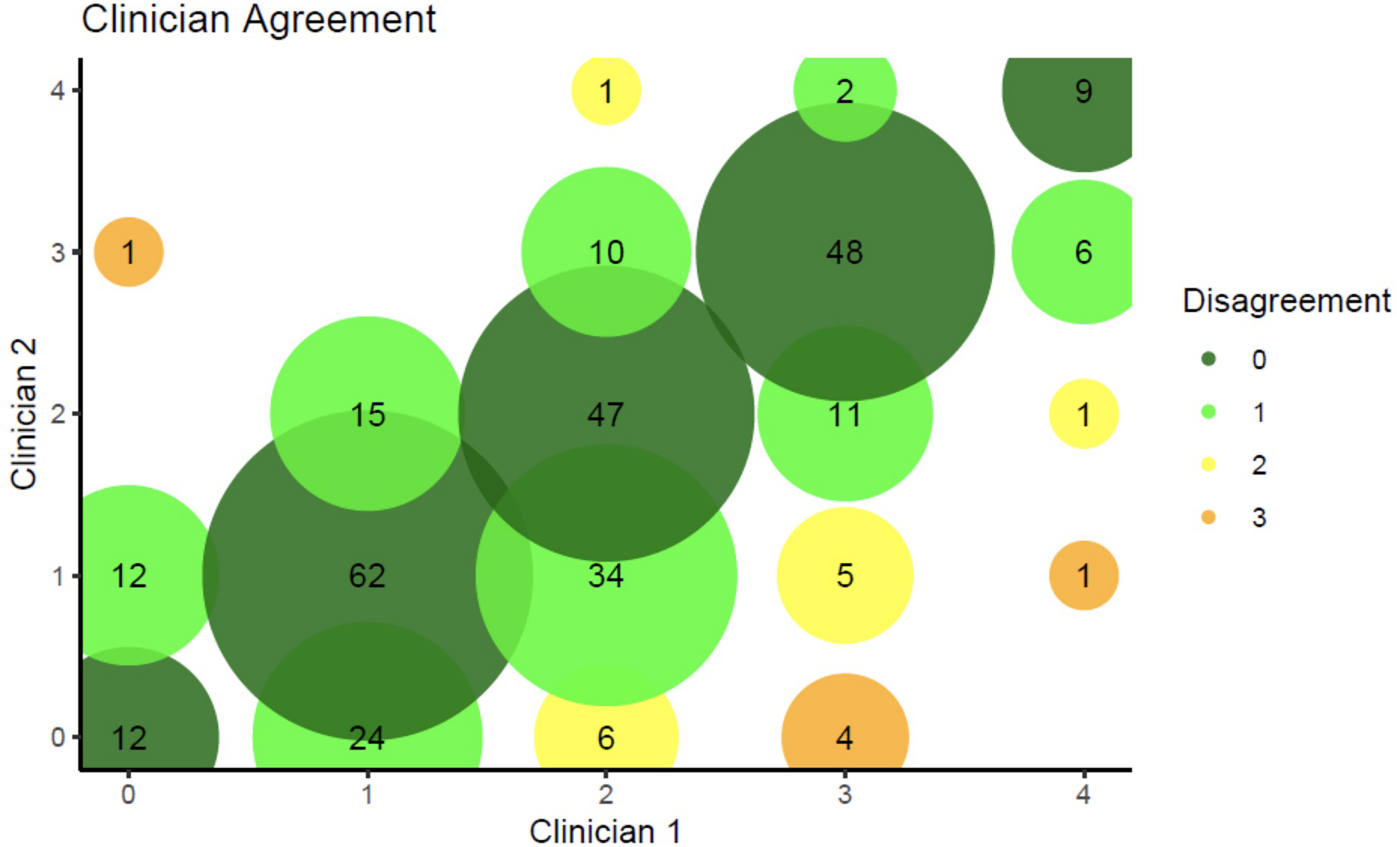
Level of agreement between the two independent clinicians. Colours represent the level of disagreement, with dark green representing complete agreement and orange representing the maximum observed disagreement of 3 levels. The area of each circle is proportional to the number of individuals with that combination of ratings from the two clinicians. For example, there are 62 participants who were rated as 1 by both clinicians, and this circle is dark green due to the complete agreement.

Using the Brodsky grading scale, 311 (79.1%) photographs were assessed by both clinicians, and all results reported hereafter (unless otherwise stated) are based on this set of 311 photographs. There was moderate agreement (*k* = 0.69, *p *< .001) between clinicians when using square-weighted κ, and weak agreement (*k* = 0.57, *p *< .001) when using equal-weighted κ (Figure 1). Of the photographs assessed, on average between the two independent clinicians, Brodsky tonsil gradings were 0 (11.4%), 1 (34.6%), 2 (27.7%), 3 (21.7%) and 4 (4.7%) (see Supplementary Table S2 and Supplementary Figure S3). Examples of Brodsky grade 1 and 4 photographs are shown in Supplementary Figure S4.

#### 3.3.3 Relationship between age and Brodsky grade

**Figure 2:**
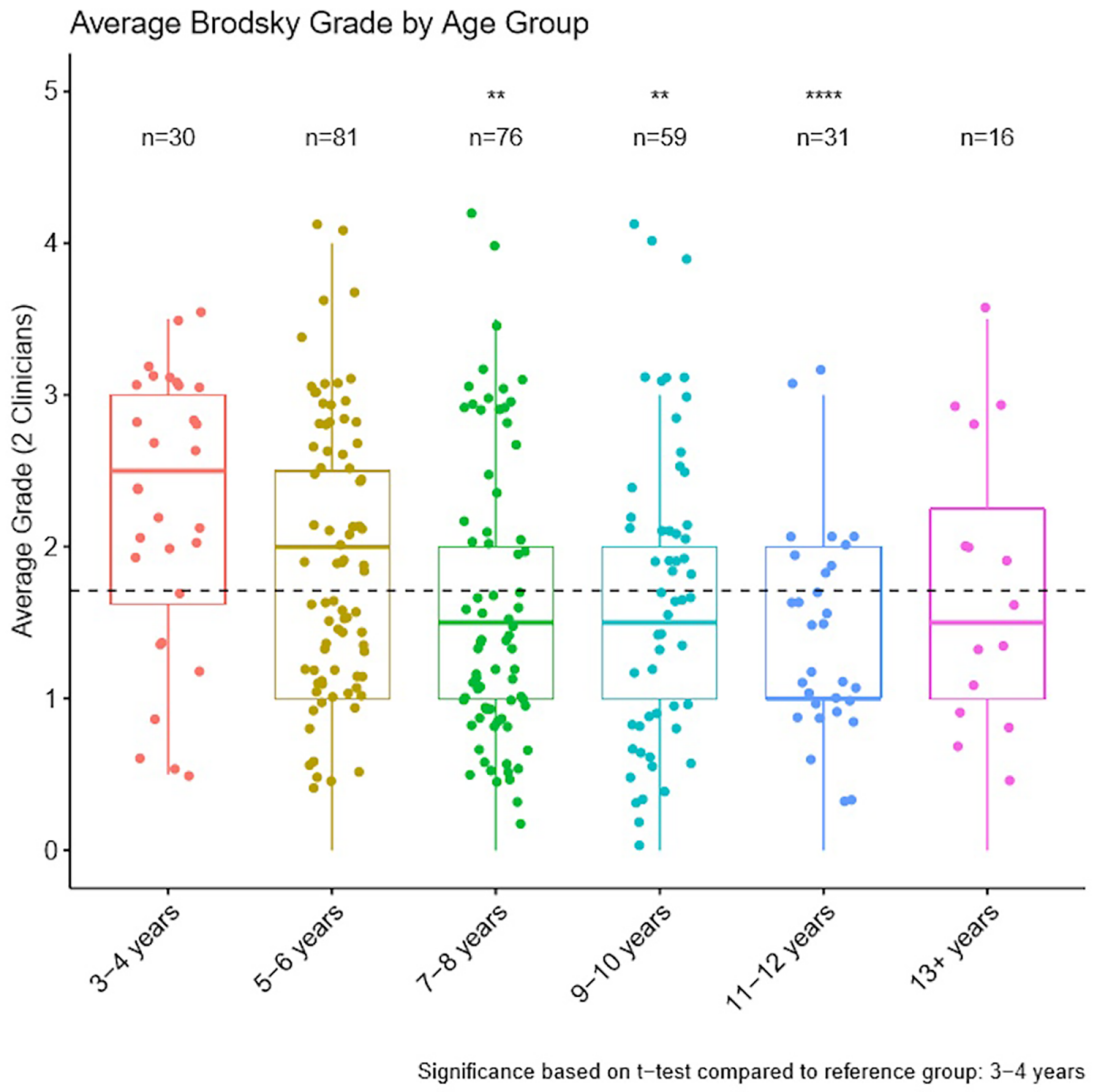
Average Brodsky tonsil grade by age assessed by two independent clinicians (median ± IQR). A dashed horizontal line displays average Brodsky grade across all age groups. **p < 0.01, ****p < 0.0001, based on *t*-test comparison to 3–4 years group.

Brodsky tonsil gradings were analysed by age group where matched demographic and grading data were available (293 participants). Figure 2 shows the frequency of tonsil grade grouped by age. Neither clinician assessed grade 4 tonsil size to any child over 10 years old. All unanimous grade 4 tonsil size ratings occurred for children aged 5–10 years.

**Figure 3:**
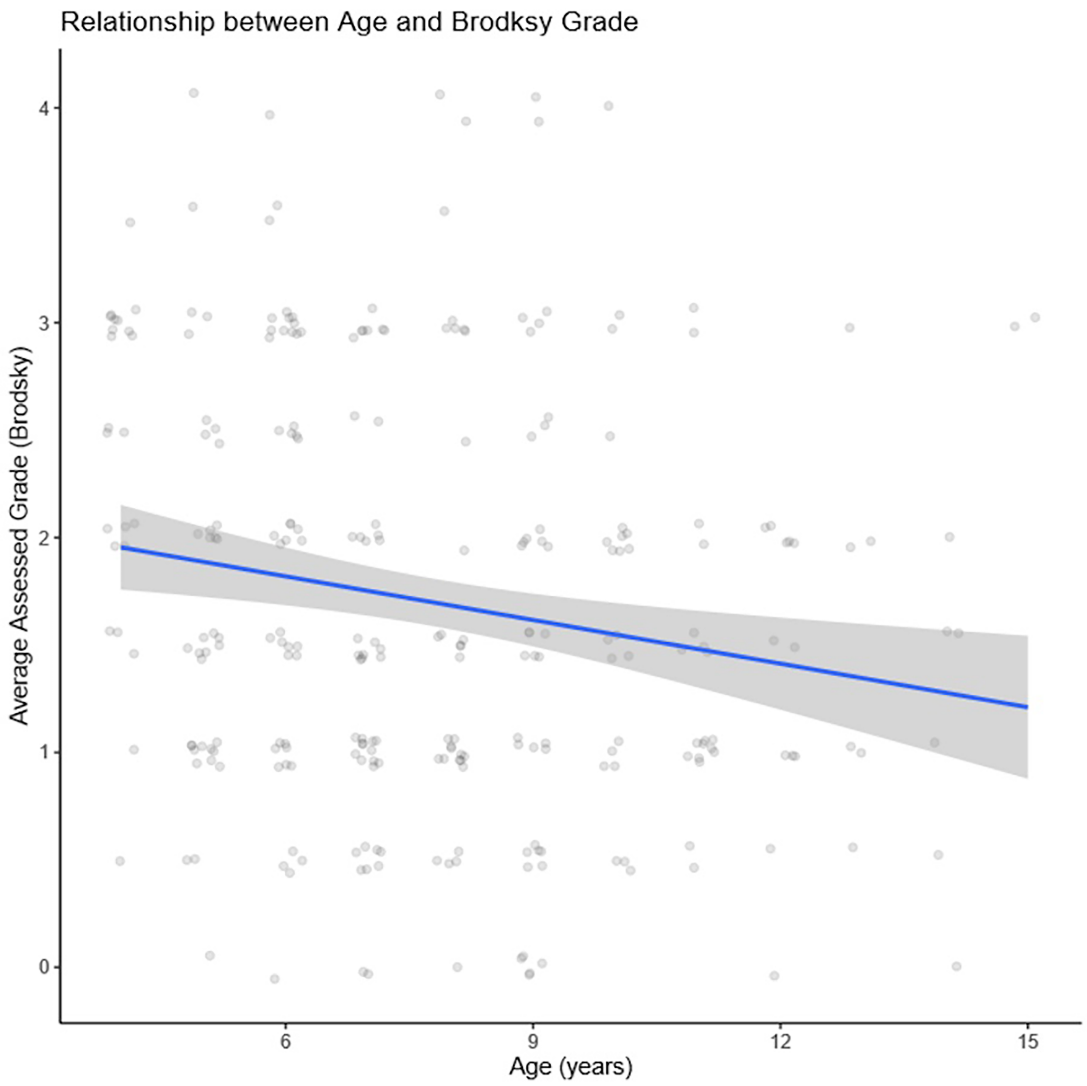
Linear regression displaying significant association between increasing age and lower grades (−0.06; 95% CI, −0.10 to −0.02; *p *= .003).

We performed a linear regression to quantify the relationship between age and average (between clinicians) assigned grade (Figure 3). Increasing age was significantly associated with lower grades (−0.06; 95% CI, −0.10 to −0.02; *p *= .003). That is, for each additional year of age, average assigned grade was expected to decrease by 0.06 which equates to a decrease of 0.66 grades from age 4–15 years.

#### 3.3.4 Signs of infection (swelling, pus, redness)

Interrater reliability was minimal to weak for clinical signs of infections identified in the photographs. There was minimal agreement (*k* = 0.32, *p *< .001) between clinicians for signs of oedema (swelling), weak agreement (*k* = 0.40, *p *< .001) for purulent exudate (pus), and minimal agreement (*k* = 0.29, *p *< .001) for erythema (redness). Clinician one assessed 391 children for tonsillitis signs and reported oedema in 49 (12.5%), exudate in 15 (3.8%) and erythema in 69 (17.6%) participants (Table 1). Clinician two assessed 393 participants and reported oedema in 57 (14.5%), exudate in 13 (3.1%) and erythema in 88 (22.4%) participants. Across all three signs of infection, clinician one more frequently classified a photograph as showing no oedema/exudate/erythema, and clinician two more frequently classified a photograph as unable to assess for oedema/exudate/erythema (see Table 1).

**Table 1:** Assessment of tonsillitis signs by two independent clinicians.

	Clinician 1 *n* *=* 391	Clinician 2 *n* *=* 393	Interrater reliability
Oedema			
Yes	49 (12.5%)	57 (14.5%)	*k* = 0.32
No	257 (65.7%)	144 (36.6%)	*p* < 0.001
Not assessable	85 (21.7%)	192 (48.9%)	* *
Exudate			
Yes	15 (3.8%)	12 (3.1%)	*k* = 0.40
No	285 (72.9 %)	184 (46.8%)	*p* < 0.001
Not assessable	91 (23.3%)	197 (50.1%)	* *
Erythema			
Yes	69 (17.6%)	88 (22.4%)	*k* = 0.29
No	240 (61.4%)	131 (33.3%)	*p* < 0.001
Not assessable	82 (21.0%)	174 (44.3%)	* *

#### 3.3.5 Sore throat symptomology (“tell us about your tonsils”)

Symptomology data were collected for 394 participants. Of those, 357 (90.6%) reported no sore throat, 33 (8.4%) reported a sore throat and four (1.0%) reported they were unsure if their throat was sore. In addition, 89 (22.5%) participants reported other potential symptoms of infection; 46 (11.7%) reported a cough, 48 (12.2%) reported a runny nose, and 22 (5.6%) participants reported two or more symptoms (Table 2). Of the 33 participants who reported a sore throat, 21 (63.6%) also reported one or more other potential symptoms of infection.

**Table 2:** Symptoms reported by participants.

Does the participant have any of these symptoms today? *n = *394	*n* (%)
Self-reported fever	4 (1.0)
Cough	46 (11.7)
Runny nose	48 (12.2)
Sore neck	3 (0.8)
Dribbling	0 (0.0)
Difficulty swallowing	3 (0.8)
Not wanting to eat or drink	2 (0.5)
Headache	6 (1.5)
Earache	3 (0.8)

#### 3.3.6 RADT (“test your tonsils”)

RADT were collected from 334 participants. Of these, 2 (0.6%) returned a positive result. These two participants reported no sore throat or infection/illness symptoms.

## 4 Discussion

This is the first study to our knowledge to recruit children at a public science event, develop a set of standardised tonsil photographs, and assess tonsillar size using photographs in lieu of the assessments typically used for real-time clinical assessment. Further, we have included comparative assessments from two clinicians of each of the available photographs to validate this innovative method. This study was feasible at a public science event and has clinical implications globally for improving comparative assessments of pharyngitis in children aged 3–15 years. This approach may have future applications in helping to improve pharyngitis assessment in remote contexts, where physicians are not always available and immediate access to assessments by clinicians or ear, nose and throat specialists may be limited.

Our results demonstrate moderate agreement between clinicians in grading tonsil size using photographs and the Brodsky Grading Scale for Tonsillar Hypertrophy. However, there was limited interrater reliability for signs of infection (swelling, redness, pus). Improvements to photo quality and therefore interrater reliability may be achievable using a single, trained clinical photographer as found in many tertiary hospitals. However, generally situations that require the use of digital images are those in which there are fewest resources and minimally trained clinicians or parents provide images that are captured, stored, and forwarded for assessment by a doctor who may be thousands of kilometres away. As such, in these situations having a single highly trained photographer may not be possible. Hence, these standardised tonsil photographs may be useful for assessing tonsillar size via telehealth, in assessing change in tonsillar size between presentations, in research or in primary prevention of ARF where clinical resources are limited. We will compare this standardised set from urban children with another study of remote living children at high risk of ARF and RHD (12).

Askarian and colleagues (15) developed a novel detection methodology for GAS throat infection utilising a smartphone, add-on gadget and post-processing algorithm, in adults, and reported an accuracy of 94% for GAS throat infection detection (15). This technology and method had not been published at the time of data collection for this study, and is limited by not including children as childhood is the primary age when pharyngitis presents. Further advancements in this type of methodology in children could be considered in future research.

The tonsillar size we observed in urban children in Australia were larger than international studies (14). Most tonsils were rated as grade 1, followed by 2 and 3, whilst grade 0 and 4 were infrequent. Akcay and colleagues using physical examination of tonsil size, identified the majority (63%) of tonsils as grade 1, followed by 2 (28%), but 3 and 4 occurred infrequently (2% and <1% respectively), in a similar age bracket to our cohort (4–17 years old) (14). The authors did not use a grade 0 rating, so we are unable to compare these results. Their study had a larger sample size (>1,000 children) than this study. The authors do not report on the average age of the cohort but note that tonsil size peaked at ages 4–8 years, with grade 4 tonsils observed only in six and seven-year-old children, which was the average age of our cohort. A limitation of our study was that real-time physical examination/grading of tonsils was not completed, so cannot be correlated with grades determined from the clinicians' assessment of these photographs. Future studies will benefit from documenting and comparing tonsil size via both methods.

In this urban population at low risk of developing ARF, we found that few (<1%) had evidence of throat infection with only two RADTs for GAS found to be positive. Low numbers of positive RADTs and grade 4 tonsil sizes were anticipated given that the data was collected from a well, community-based cohort. The two positive RADT results were from participants who did not report a sore throat or other symptoms of infection. As the children had no symptoms of infection, the corresponding tonsillar photographs were not individually reported. It is possible that the positive tests were detecting GAS carriage, however the RADT is validated only for detecting GAS pharyngitis and not carriage—and therefore would not usually have been performed in an asymptomatic child. As throat swabs for GAS culture were not collected, confirmation of GAS identification in the throat remains a limitation of the study, and these two positive tests may have been false positives. Future prospective studies in remote Indigenous cohorts will collect culture and serological tests to assess the presence of active or previous GAS infection and potential GAS carriage (12). Analysis of the subset of tonsillar photographs from children who presented with sore throats was not performed but could be considered in future studies.

Other limitations of the study included the rapid pace of data collection which contributed to varying rates of participation (and therefore denominators) in each of the three main data collection activities and results. Multiple research staff, while trained in the standard operating procures, may have contributed to the varying quality of photographs, with some staff more familiar with interacting with children than others. While the public science setting of this initial study allowed for rapid completion of data collection, across a broad cross-section of healthy children, more regimented data collection in future studies could improve the quality of photographs and interrater reliability. Observationally it was noted that for some children a photograph with a clear view to the back of the throat was not possible to collect (usually due to obstruction by the tongue or the child being unable to open their mouth wide enough despite prompting). This is likely to have contributed to the portion of photographs deemed not able to be graded by the independent clinicians. The sample size of over 400 participants in an urban setting public science event may not be generalisable to other populations and clinical settings, and further studies are needed. Further, tonsillar hypertrophy is not diagnostic of GAS pharyngitis and, thus, the photographs should not be used alone in diagnosis. Future studies could compare this set of photographs from healthy children to those with GAS pharyngitis to better validate and assess the clinical utility of this set of tonsillar photographs.

## 5 Conclusions

We report the first collection of tonsil photographs in children globally. We successfully assessed tonsil size using a standardised method carried out within an urban-living cohort of healthy children in Australia. The learnings from this study will be applied in future studies and these results compared to those from future studies involving children living in remote Indigenous communities in Australia and other populations globally at high risk of rheumatic fever. This will help progress our research towards improved primary prevention strategies to end RHD in remote-living Indigenous populations of Australia and align with the WHO resolution to prioritise rheumatic fever and RHD as global health problems (25).

## Data Availability

The raw data supporting the conclusions of this article will be made available by the authors, without undue reservation.
