# Supplementary material for: Tonsils at Telethon: developing a standardised collection of tonsil photographs for group A streptococcal (GAS) research
Source: Front Pediatr. 2024 Apr 25;12:1367060. doi: 10.3389/fped.2024.1367060 (PMC11079290; doi:10.3389/fped.2024.1367060)
Supplement: Supplementary file 1 [file Datasheet1.pdf]

frontiers

Supplementary Material

Tonsils at Telethon: developing a standardized collection of tonsil photographs for group A streptococcal (GAS) research

Marianne J Mullane[1], Hannah M Thomas[1], Jonathan R Carapetis[1,2], Catalina Lizama[1], Wesley Billingham[1], Matthew N Cooper[1], Christine Everest[1], Claudia R Sampson[1,2], Nelly Newall[1], Sarah Pearce[1], Francis Lannigan[2], Eamonn McNulty[2], Rebecca Cresp[2], Ariel O Mace[1,2], Tina Barrow[1], Asha C Bowen[1,2]

[1]Wesfarmers Centre of Vaccines and Infectious Diseases, Telethon Kids Institute, University of Western Australia, Perth, Western Australia, Australia

[2]Perth Children's Hospital, Perth, Western Australia, Australia

*** Correspondence:**
Hannah Thomas
Hannah.Thomas@telethonkids.org.au

1.1 Supplementary Figures

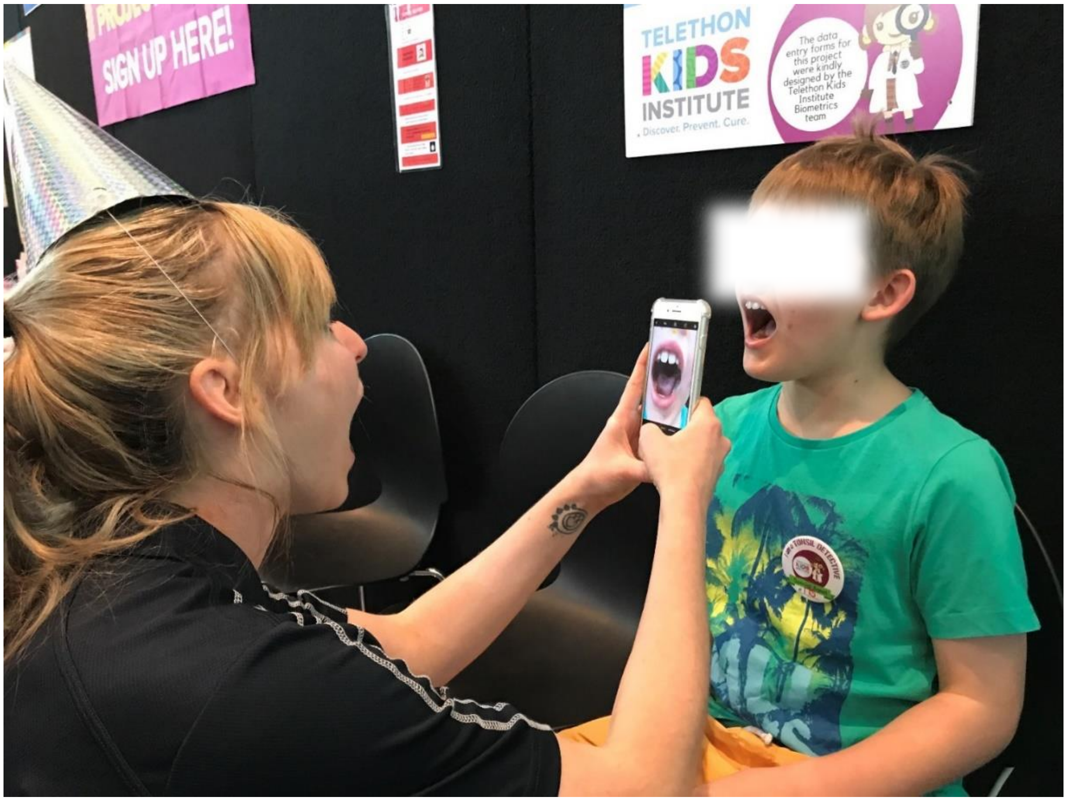

Supplemental Figure 1. Children were invited to “take a tonsil selfie” with researchers using the iPhone 7 Plus and study tonsil photography standard operating procedures.

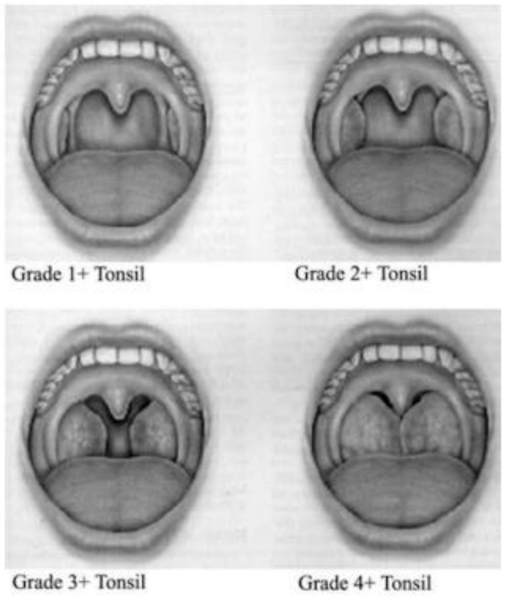

**Grade 0**	Tonsils within the tonsillar fossa
**Grade 1**	Tonsils just outside tonsillar fossa, occupy 25% of oropharyngeal width
**Grade 2**	Tonsils occupy 26-50% oropharyngeal width
**Grade 3**	Tonsils occupy 51-75% of oropharyngeal width
**Grade 4**	Tonsils occupy ≥75% oropharyngeal width

Adapted from: Brodsky L. Modern assessment of tonsils and adenoids. Pediatr Clin North Am. 1989 Dec;36(6):1551-69.

Supplemental Figure 2. Brodsky Grading Scale of Tonsillar Hypertrophy. From: Brodsky L. Modern Assessment of tonsils and adenoids. Pediatr Clin North Am. 1989 Dec;36(6):1551-1569.

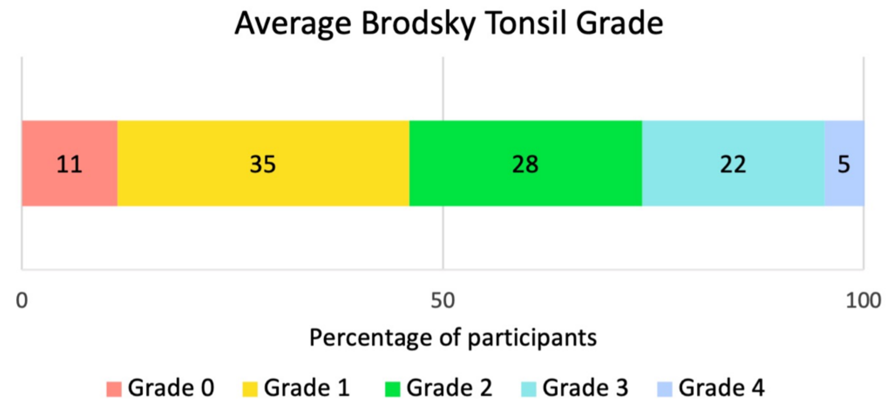

Supplemental Figure 3. Average Brodsky tonsil grade assessed by two independent clinicians.

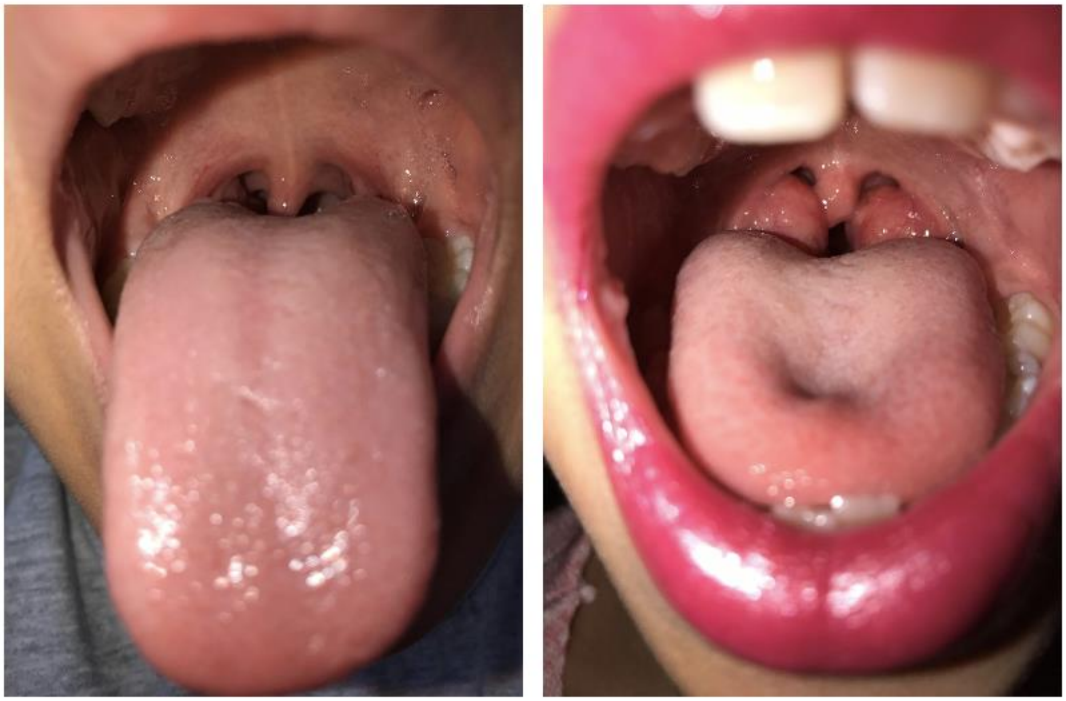

Supplemental Figure 4. Example tonsil photographs showing Brodsky grade for tonsil size. The photograph on the left shows grade 1 (tonsils occupy 25% of the oropharyngeal width). The photograph on the right shows grade 4 (tonsils occupy ≥75% of the oropharyngeal width).

1.2 Supplementary Tables

Supplemental Table 1. Participant demographics.

Demographics	Number	%
Median (IQR) age; *n = 393*	7.4 (5.9-9.7)	
Gender; *n = 426*		
Male	156	36.6
Female	239	56.1
Other	0	0.0
Missing	31	7.3
Ethnicity; *n = 426*		

Indigenous	8	1.9
Caucasian	261	61.3
Pacific Islander	5	1.2
Other	99	23.2
Unknown	14	3.3
Prefer not to answer	3	0.7
Missing	48	11.3

Supplemental Table 2. Brodsky tonsil grade assessed by two independent clinicians.

**Grade**	**Clinician 1** *n = 311*	**Clinician 2** *n = 311*	**Average** *n = 311*
**0**	25 (8.0%)	46 (14.8%)	36 (11.4%)
**1**	101 (32.5%)	114 (36.7%)	108 (34.6%)
**2**	98 (31.5%)	74 (23.8%)	86 (27.7%)
**3**	70 (22.5%)	65 (20.9%)	68 (21.7%)
**4**	17 (5.5%)	12 (3.9%)	15 (4.7%)
